# The Doctors of Georgia

**Published:** 1881-11-20

**Authors:** 


### *THE DOCTORS OF GEORGIA*

Should bear in mind that the new Medical Law of the State requires them to go before the County Clerk, show their diplomas or licenses, and register their names, on or before the 1st of December next, (1881).

The Pharmaceutical Board of Georgia, under the late act of the legislature, will hold its first meeting for examining applicants for license on the 13th of December, in Atlanta.

